# Adding high-intensity interval training to conventional training modalities: optimizing health-related outcomes during chemotherapy for breast cancer: the OptiTrain randomized controlled trial

**DOI:** 10.1007/s10549-017-4571-3

**Published:** 2017-11-14

**Authors:** Sara Mijwel, Malin Backman, Kate A. Bolam, Anna Jervaeus, Carl Johan Sundberg, Sara Margolin, Maria Browall, Helene Rundqvist, Yvonne Wengström

**Affiliations:** 1 Department of Physiology and Pharmacology, Karolinska Institutet, Stockholm, Sweden; 2 Department of Neurobiology, Care Sciences and Society, Karolinska Institutet, Huddinge, Sweden; 3 School of Human Movement and Nutrition Sciences, The University of Queensland, Brisbane, Australia; 4 Department of Learning, Informatics, Management and Ethics, Karolinska Institutet, Stockholm, Sweden; 5 Department of Clinical Science and Education, Södersjukhuset, Karolinska Institutet, Stockholm, Sweden; 6 Department of Oncology, Stockholm South General Hospital, Stockholm, Sweden; 7 School of Health and Education, University of Skövde, Skövde, Sweden; 8 Department of Cell and Molecular Biology, Karolinska Institutet, Stockholm, Sweden; 9 Theme Cancer, Karolinska University Hospital, Stockholm, Sweden

**Keywords:** High-intensity interval training, Concurrent training, Breast cancer, Chemotherapy, Symptom burden, Health-related quality of life

## Abstract

**Purpose:**

Exercise training is an effective and safe way to counteract cancer-related fatigue (CRF) and to improve health-related quality of life (HRQoL). High-intensity interval training has proven beneficial for the health of clinical populations. The aim of this randomized controlled trial was to compare the effects of resistance and high-intensity interval training (RT–HIIT), and moderate-intensity aerobic and high-intensity interval training (AT–HIIT) to usual care (UC) in women with breast cancer undergoing chemotherapy. The primary endpoint was CRF and the secondary endpoints were HRQoL and cancer treatment-related symptoms.

**Methods:**

Two hundred and forty women planned to undergo chemotherapy were randomized to supervised RT–HIIT, AT–HIIT, or UC. Measurements were performed at baseline and at 16 weeks. Questionnaires included Piper Fatigue Scale, EORTC-QLQ-C30, and Memorial Symptom Assessment Scale.

**Results:**

The RT–HIIT group was superior to UC for CRF: total CRF (*p* = 0.02), behavior/daily life (*p* = 0.01), and sensory/physical (*p* = 0.03) CRF. Role functioning significantly improved while cognitive functioning was unchanged for RT–HIIT compared to declines shown in the UC group (*p* = 0.04). AT–HIIT significantly improved emotional functioning versus UC (*p* = 0.01) and was superior to UC for pain symptoms (*p* = 0.03). RT–HIIT reported a reduced symptom burden, while AT–HIIT remained stable compared to deteriorations shown by UC (*p* < 0.01). Only RT–HIIT was superior to UC for total symptoms (*p* < 0.01).

**Conclusions:**

16 weeks of resistance and HIIT was effective in preventing increases in CRF and in reducing symptom burden for patients during chemotherapy for breast cancer. These findings add to a growing body of evidence supporting the inclusion of structured exercise prescriptions, including HIIT, as a vital component of cancer rehabilitation.

**Trial registration:**

Clinicaltrials.gov Registration Number: NCT02522260.

## Introduction

Adjuvant chemotherapy improves survival and decreases the risk of recurrence of breast cancer [1]. However, the treatment is associated with considerable side effects that negatively impact an individual’s health-related quality of life (HRQoL) [2–4]. Cancer-related fatigue (CRF) is often reported as the most debilitating of all symptoms experienced during chemotherapy [4, 5], and has been described as a cancer treatment toxicity that can last for several years into survivorship [6].

Exercise training has been shown to improve HRQoL [7] and has been established as an effective intervention to manage CRF [8], being superior to both pharmacological and psychological interventions [9]. While both aerobic exercise and resistance exercise alone have shown positive health effects for women with breast cancer [10], cancer-specific exercise guidelines recommend that people with breast cancer engage in a combination of aerobic and resistance exercise [11].

There is increasing evidence highlighting the benefits of shorter bursts of high-intensity exercise for clinical populations [12, 13]. The evidence shows not only improvements in cardiorespiratory fitness compared to moderate-intensity aerobic training, but also added benefits on quality of life [14], mood state [15, 16], and cognitive health [17] and increases endorphin release in brain areas associated with controlling emotion and pain [18]. Pilot studies have shown that patients with cancer can perform HIIT safely [19–21]. Given that one major barrier to perform physical exercise is lack of time and motivation [22], HIIT during chemotherapy may be favorable for optimizing health outcomes. We hypothesize that combining HIIT with conventional exercise provide added benefits on CRF, HRQoL, and symptom burden. This is the first large RCT to incorporate this emerging training modality in patients with breast cancer during chemotherapy. The aim of this study was to compare the effects of two training interventions: 1) resistance and high-intensity interval training (RT–HIIT), and 2) moderate-intensity aerobic and high-intensity interval training (AT–HIIT), to a control group receiving usual care (UC) on CRF, HRQoL, and symptoms in women with breast cancer undergoing chemotherapy.

## Methods

### Study design

The OptiTrain randomized controlled trial (NCT02522260, www.clinicaltrials.gov) was an in-clinic randomized controlled exercise trial [23]. Participants were randomly allocated to either group 1: RT–HIIT, group 2: AT–HIIT, twice a week for 16 weeks, or group 3: control group receiving UC. The primary endpoint CRF and the secondary endpoints HRQoL and symptom/symptom burden were measured 1 week prior to the participants’ second chemotherapy session and at 16 weeks. The intervention groups (RT–HIIT and AT–HIIT) commenced the exercise training 3 days after the second chemotherapy session. The rationale for performing baseline assessments after the first round of chemotherapy was the limited time to perform ECG and baseline testing between randomization and the first chemotherapy session.

### Setting and participants

Recruitment took place at two different oncology clinics in Stockholm, Sweden from March 2013 to July 2016 and 628 patients were eligible and invited to participate in the study by the referring oncologists. Two hundred and forty women volunteered to participate in the study. Eligibility criteria were as follows: women (i) aged 18–70 years, (ii) diagnosed with I–IIIa stage breast cancer, and (iii) planned to receive adjuvant chemotherapy (consisting of anthracyclines, taxanes, or a combination of the two). Participants were excluded if they had advanced disease, heart or lung disease, cognitive dysfunction, or did not speak or understand the Swedish language. Interested participants answered a questionnaire about their cardiovascular health history [24] and underwent a resting echocardiogram before enrollment to rule out cardiac pathologies. Ethical approval was obtained from the Regional Ethical Review ‎Board in Stockholm Sweden (Dnr 2012/1347-31/1, 2012/1347-31/2, 2013/7632-32, and 2014/408-32) and all participants gave written informed consent.

### Randomization and blinding

The participants were randomly allocated to RT–HIIT, AT–HIIT, or UC, by the Clinical Studies Unit at Karolinska University Hospital, using a random assignment computer program at a 1:1:1 ratio blinded to the research team, prior to the first assessment. Participants, exercise supervisors, and outcome assessors were not blinded to group allocation.

### Exercise intervention

The RT–HIIT and AT–HIIT groups undertook the exercise sessions in an exercise clinic twice weekly for 16 weeks. Exercise session duration was approximately 60 min, supervised by an exercise physiologist or an oncology nurse [23]. The program was extended for participants with delays in chemotherapy (RT–HIIT, *n* = 8, range: 15–35 days; AT–HIIT, *n* = 5, range: 13–32 days). All exercise sessions began with a 5-min warm up on a cycle ergometer or treadmill at a rating of perceived exertion (RPE) of 10–12 on the Borg scale [25], and ended with a 10-min cool down of dynamic muscle stretching. The RT–HIIT group performed both resistance and high-intensity interval aerobic exercise at each session. The resistance training component consisted of exercises targeting the major muscle groups using weight stack training equipment, participants’ body mass, free weight dumbbells or barbells. Exercises included leg press, biceps curls, squat jumps, triceps extensions, lunges, bench press, sit-ups or Russian-weighted abdominal twist, shoulder press, and prone-lying back extensions. Participants performed two to three sets of 8–12 repetitions at an initial intensity of 70% of their estimated one repetition maximum (1-RM) using a prediction equation [26], progressing to 80% of 1-RM when more than 12 repetitions could be performed. To ensure overload, new estimated 1-RM tests were performed when participants could lift more than 12 repetitions of their 80% 1-RM. The aerobic component of the RT–HIIT program consisted of 3 × 3-min bouts of high-intensity interval aerobic exercise on a cycle ergometer at a rating of perceived exertion of 16–18 on the Borg scale interspersed with one min of low-intensity active recovery. The AT–HIIT group commenced with 20 min of moderate-intensity, continuous aerobic exercise at an RPE of 13–15 on a cycle ergometer, elliptical ergometer, or treadmill. This was followed by the same high-intensity interval exercise training as RT–HIIT. The UC group was given written information about physical activity at the initiation of the intervention period about exercise recommendations for patients with cancer according to American College of Sports Medicine guidelines [11].

### Outcome measures

All outcome assessments took place at baseline and at 16 weeks.

#### Primary outcome

CRF was self-assessed using the Swedish version of the 22-item Piper Fatigue Scale (PFS) which has been validated in the Swedish population [27], covering four dimensions of fatigue: behavioral/daily life (6 items), sensory/physical (5 items), cognitive (6 items), and affective/emotional meaning (5 items). Each item is composed of a scale from zero to ten, with zero indicating “no fatigue.” Scores were calculated according to recommended scoring procedures [28]. The instrument is reliable and has been used extensively in patients with breast cancer even though sensitivity to change is yet to be established [27].

#### Secondary outcomes

The 30-item EORTC-QLQ-C30 questionnaire (version 3.0) was used to assess HRQoL [29]. This questionnaire incorporates a global HRQoL score, five functional subscales, and nine symptoms, including general CRF. Despite lacking different dimensions of CRF, the EORTC-QLQ-C30 is valid and reliable to assess CRF [30], and is sensitive to change when used for patients with cancer during chemotherapy [31]. Symptoms and symptom burden were assessed using the 32-item Memorial Symptom Assessment Scale (MSAS) [32, 33]. The scale includes occurrence, frequency, severity, and distress associated with each symptom using four- and five-point rating scales. Total symptom score was calculated as the average of all 32 symptoms, and symptom burden (the degree to which the symptoms affect daily life) was calculated as the average of the frequency of four psychological symptoms and the distress associated with six physical symptoms. The physical and psychological symptom subscales were calculated as the average of the frequency, severity, and distress associated with 12 physical symptoms, and six psychological symptoms, respectively.

To explore if baselines values influenced change over the intervention, sub-analyses were performed for the outcomes CRF, global HRQoL, symptom burden, and total symptoms. Cut-off values at baseline were categorized as CRF (score > 0) and low global HRQoL according to a validated cut-off score (score < 80) [34]. For symptoms, the cut-off was chosen based on participants’ mean score at baseline: high symptom burden (score > 0.79), and high total symptom (score > 0.66). No statistics were performed for these variables.

#### Additional measures

Demographic and medical information were collected through questionnaires and extracted from patients’ electronic medical records. Objectively measured activity patterns were assessed at baseline by an accelerometer (GT3X ActiGraph^®^ Corp, Pensacola, Florida, USA), which the participants were instructed to wear on an elastic belt over their right hip during all waking hours for seven consecutive days. Data were downloaded using the ActiLife v.6.10.1 software and analyzed using validated wear-time specifications and cut-offs for adults [35].

Attendance was calculated as the mean of the individual percentages (attended exercise sessions divided by the total number of sessions). Adherence to the exercise regimen was calculated as the number of patients who successfully completed 90% of the exercise sessions according to plan (i.e., intensity and duration), divided by the total number of patients in the intervention groups.

#### Adverse events

At each exercise session, participants reported any adverse events to the exercise supervisors and these were recorded in the participants’ log records.

### Statistical methods

With CRF as the primary outcome measure, a sample size of 65 participants per group was required, based on an effect size of 0.53 and 80% power at an alpha level of 0.05 (two-tailed). With an expected attrition rate of 20%, 80 participants were required per group. Data were analyzed using the IBM^®^ SPSS^®^ version 22 statistical package for Windows. Analyses included standard descriptive statistics, one-way analysis of variance (ANOVA), and analysis of covariance (ANCOVA), controlling for baseline scores, chemotherapy treatment (taxanes/no taxanes), and menopausal status. Where appropriate, the Bonferroni post hoc procedure for multiple comparisons was used to locate the source of significant differences. Data not normally distributed were log-transformed prior to ANCOVA analyses. RM-ANOVA or the Wilcoxon signed rank test was applied for within-group analyses (within-group results shown in Tables 2, 3, and 4). Clinically important changes were estimated as standardized effect sizes (ES) where between-group differences of pre- and post-intervention means were divided by the pooled baseline standard deviation [36]:

$$\frac{{(M_{{{\text{post}} . {\text{T}}}} - M_{{{\text{pre}} . {\text{T}}}} ) - (M_{{{\text{post}} . {\text{C}}}} - M_{{{\text{pre}} . {\text{C}}}} )}}{{SD_{{{\text{pooled}}\;{\text{pre}}}} }}.$$

According to Cohen’s guidelines, effect sizes with scores of 0.2–0.5, 0.5–0.8, and > 0.8 were considered small, medium, and large effects, respectively [37]. An intention-to-treat analysis (ITT) approach was used for the analyses, with missing values from the post-assessment being replaced using the expectation maximization method [38]. Participants that dropped out after baseline assessment and did not complete questionnaires at baseline were excluded from the analysis (*n* = 2). The expectation–maximization algorithm was based on group change. All tests were two-tailed and statistical significance was set at *p* < 0.05.

## Results

**Fig. 1:**
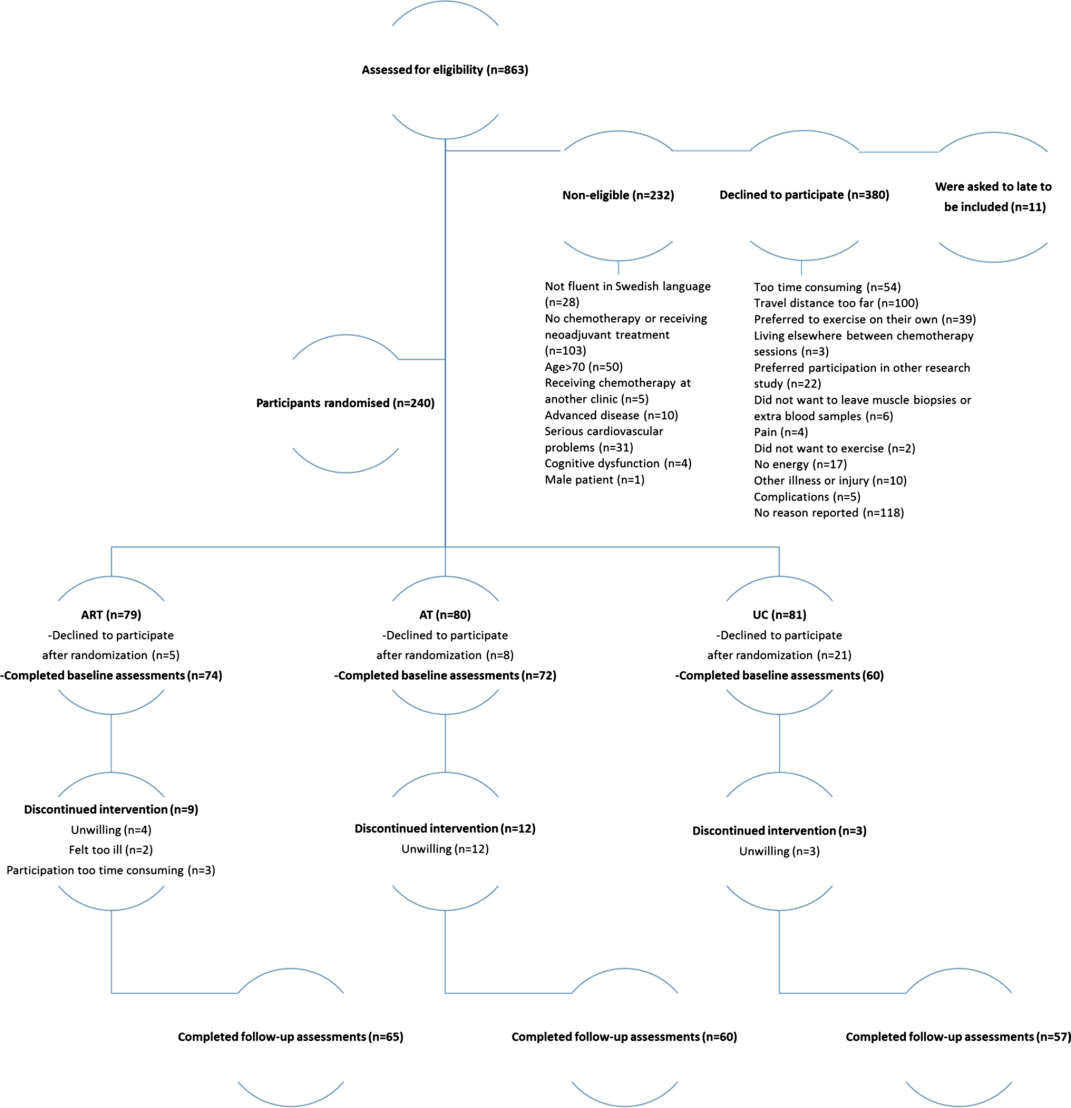
CONSORT diagram. *RT–HIIT* resistance and high-intensity interval training, *AT–HIIT* moderate-intensity aerobic and high-intensity interval training, *UC* usual care

In total, 240 women agreed to participate, and 182 participants completed the baseline and follow-up testing. Post-assessment data were imputed for participants (*n* = 22) who had completed baseline assessment for all outcomes. The main reasons for declining to participate after randomization were too time consuming, feeling ill, or did not want to be randomized to the UC group. The CONSORT study flow chart is shown in Fig. 1. There were no significant differences at baseline between groups regarding participant characteristic data (Table 1). Immediately following randomization, fourteen participants dropped out, while ten percent of the participants dropped out during the intervention. There were no significant differences in participant characteristics between those who withdrew and participants who completed the study. Attendance rates for participants in the RT–HIIT and AT–HIIT groups were 68 and 63%, respectively, and adherence to the training program was 83% in the RT–HIIT group and 75% in AT–HIIT group.

**Table 1:** Participant characteristics at baseline (mean ± SD)

	RT–HIIT *n* = 74	AT–HIIT *n* = 72	UC *n* = 60
	Mean ± SD	Mean ± SD	Mean ± SD
Age (years)	52.7 ± 10.3	54.4 ± 10.3	52.6 ± 10.2
Body mass (kg)	68.7 ± 11.3	67.7 ± 13.0	69.1 ± 11.0
Height	165.7 ± 6.7	165.3 ± 6.6	166.4 ± 7.0
MVPA (min/day)	79.0 ± 25.0	71.0 ± 31.0	68.0 ± 31.0
SED (min/day)	530.0 ± 70.0	544.0 ± 60.0	548.0 ± 67.0

	n (%)	n (%)	n (%)
Married or partnered	60.6	59.7	69.5
Education level completed			
Primary school	16.9	16.2	17.0
Secondary school	15.5	19.1	17.0
Tertiary education	67.6	64.7	66.0
Current smoker	4.3	5.9	5.2
Employed	74.6	86.8	79.7
Menopausal status			
Premenopausal	47.3	36.1	38.3
Postmenopausal	51.4	63.9	61.7
Not known	1.4	0.0	0.0
Tumor profile			
Triple negative	14.9	11.0	16.7
HER2+, ER+, PR+	16.2	15.1	5.0
HER2+, ER−, PR−	0.0	11.0	10.0
HER2−, ER+, PR+	54.1	46.6	53.3
HER2−, ER+, PR−	8.1	12.3	8.3
HER2+, ER+, PR−	5.4	4.1	5.0
HER2−, ER−, PR+	1.4	0.0	1.7
Chemotherapy regimen			
Anthracycline	39.3	37.0	40.0
Taxane	2.7	5.5	0.0
Anthracycline + taxane	37.8	37.0	33.3
Anthracycline + taxane + herceptin	18.9	20.6	25.0
Anthracycline + herceptin	1.4	0.0	1.7

*RT–HIIT* resistance and high-intensity interval training, *AT–HIIT* moderate-intensity aerobic and high-intensity interval training, *UC* usual care, *SD* standard deviation, *MVPA* objectively measured moderate- to vigorous-intensity physical activity, *SED* objectively measured sedentary behavior

### Changes is CRF

Following the intervention, CRF assessed by the Piper Fatigue Scale increased significantly in the UC group and was significantly different from maintained levels found for RT–HIIT: total CRF (ES = − 0.51), behavior/daily life CRF (ES = − 0.62), and sensory/physical CRF (ES = − 0.47) (Table 2). Similarly, CRF assessed by the EORTC-QLQ-C30 showed a significant increase for UC that was significantly different from the unchanged CRF for RT–HIIT (ES = − 0.61) and for AT–HIIT (ES = − 0.47) (Table 3).

**Table 2:** Cancer-related fatigue (PFS) outcome variables and change over 16 weeks

	Baseline mean ± SD			16 weeks mean ± SD			Adjusted mean change Mean (95% CI)			*p* value^†^	Effect size	
	RT–HIIT *n* = 74	AT–HIIT *n* = 70	UC *n* = 60	RT–HIIT *n* = 74	AT–HIIT *n* = 70	UC *n* = 60	RT–HIIT vs UC	AT–HIIT vs UC	RT–HIIT vs AT–HIIT		RT–HIIT vs UC	AT–HIIT vs UC
Total CRF	3.09 ± 3.17	2.10 ± 2.63	2.30 ± 2.81	3.16 ± 2.92	3.16 ± 2.61*	3.94 ± 2.95*	− 1.17 (− 2.18 to − 0.16)*	− 0.72 (− 1.73 to 0.30)	− 0.46 (− 1.43 to 0.52)	0.02	− 0.51	− 0.26
Sensory/physical CRF	3.24 ± 3.23	2.27 ± 2.87	2.64 ± 3.15	3.31 ± 3.07	3.48 ± 2.96*	4.25 ± 3.21*	− 1.22 (− 2.33 to − 0.11)*	− 0.64 (− 1.76 to 0.49)	− 0.58 (− 1.66 to 0.49)	0.03	− 0.48	− 0.13
Behavior/daily life CRF	3.10 ± 3.39^ϕ^	1.87 ± 2.57	2.04 ± 2.79	3.07 ± 3.20	2.99 ± 2.74*	3.98 ± 3.19*	− 1.46 (− 2.55 to − 0.37)*	0.96 (− 2.05 to 0.14)	− 0.50 (− 1.56 to 0.55)	< 0.01	− 0.62	− 0.30
Emotional/affective CRF	3.28 ± 3.43	2.37 ± 2.98	2.45 ± 3.01	3.48 ± 3.21	3.72 ± 3.04*	4.21 ± 3.11*	− 1.10 (− 2.26 to 0.06)	− 0.49 (− 1.66 to 0.68)	− 0.61 (− 1.73 to 0.51)	0.07	− 0.46	− 0.13
Cognitive CRF	2.79 ± 2.95	1.88 ± 2.51	2.15 ± 2.70	2.85 ± 2.74	2.60 ± 2.25*	3.41 ± 2.78*	− 0.89 (− 1.81 to 0.03)	− 0.70 (− 1.63 to 0.23)	− 0.19 (− 1.08 to 0.70)	0.05	− 0.42	− 0.08

*RT–HIIT* resistance and high-intensity interval training, *AT–HIIT* moderate-intensity aerobic and high-intensity interval training, *UC* usual care, *PFS* Piper Fatigue Scale, *CRF* cancer-related fatigue

^†^ *p* values are for the interaction (ANCOVA adjusted for baseline values, chemotherapy, and menopausal status), * *p* < 0.05 pre- to post-intervention, ^ϕ^ significant difference at baseline between RT–HIIT and AT–HIIT

**Table 3:** Health-related quality of life (EORTC-QLQ-C30) outcome variables and change over 16 weeks

	Baseline Mean ± SD			16 weeks Mean ± SD			Adjusted mean change Mean (95% CI)			*p* value^†^	Effect size	
	RT–HIIT *n* = 74	AT–HIIT *n* = 70	UC *n* = 60	RT–HIIT *n* = 74	AT–HIIT *n* = 70	UC *n* = 60	RT–HIIT vs UC	AT–HIIT vs UC	RT–HIIT vs AT–HIIT		RT–HIIT vs UC	AT–HIIT vs UC
Global health status/QoL	63.56 ± 24.97	66.67 ± 20.90	68.84 ± 21.66	63.68 ± 19.11	63.48 ± 19.06	59.91 ± 19.03*	5.47 (− 1.74 to 12.67)	4.67 (− 2.60 to 11.94)	0.80 (− 6.12 to 7.72)	0.15	0.40	0.29
Physical functioning	89.38 ± 14.67	89.98 ± 11.42	88.25 ± 16.58	85.88 ± 15.87*	85.37 ± 14.49*	77.49 ± 19.65*	7.38 (1.77 to 12.98)*	6.92 (1.24 to 12.60)*	0.46 (− 4.94 to 5.86)	< 0.01	0.49	0.48
Emotional functioning	67.85 ± 25.80	74.42 ± 18.94	74.47 ± 24.32	72.24 ± 22.23	78.91 ± 15.84*	70.10 ± 25.60	6.09 (− 0.96 to 13.14)	8.81 (1.72 to 15.90)*	− 2.72 (− 9.51 to 4.07)	0.01	0.34	0.40
Role functioning	59.91 ± 34.72	67.61 ± 30.55	69.76 ± 28.07	71.59 ± 27.25*	70.77 ± 25.06	54.84 ± 33.03*	20.30 (9.38 to 31.21)*	17.07 (6.09 to 28.04)*	3.23 (− 7.24 to 13.71)	< 0.01	0.81	0.64
Cognitive functioning	77.00 ± 26.05	81.39 ± 20.60	78.78 ± 25.09	78.29 ± 20.13	78.88 ± 18.99	70.76 ± 26.65*	8.63 (1.11 to 16.14)*	6.68 (− 0.92 to 14.28)	1.95 (− 5.30 to 9.19)	0.02	0.35	0.27
Social functioning	64.98 ± 29.91	72.91 ± 24.26	71.65 ± 29.63	62.32 ± 46.87	71.33 ± 23.94	63.30 ± 27.67*	2.45 (− 11.12 to 16.01)	7.44 (− 6.24 to 21.11)	− 4.99 (− 18.07 to 8.09)	0.40	0.19	0.27
Fatigue	39.74 ± 29.88	35.54 ± 23.28	34.08 ± 25.37	37.18 ± 23.70	38.59 ± 23.51	48.49 ± 24.88*	− 13.45 (− 22.41 to − 4.49)*	− 10.86 (− 19.00 to − 1.81)*	− 2.59 (− 11.21 to 6.02)	0.01	− 0.61	− 0.47
Nausea and vomiting	12.87 ± 16.37	12.99 ± 18.15	8.27 ± 17.16	5.02 ± 10.10*	6.02 ± 11.39*	7.79 ± 20.37	− 3.93 (− 9.60 to 1.74)	− 3.21 (− 8.95 to 2.53)	− 0.72 (− 6.15 to 4.71)	0.21	− 0.44	− 0.37
Pain	21.62 ± 24.82	15.48 ± 22.40	17.32 ± 26.24	21.01 ± 24.06	17.35 ± 19.08	27.95 ± 30.20*	− 8.32 (− 17.67 to 1.04)	− 10.16 (− 19.63 to 0.69)	1.84 (− 7.22 to 10.91)	0.025	− 0.44	− 0.36
Dyspnoea	25.11 ± 27.52	22.25 ± 22.46	28.45 ± 25.70	35.17 ± 28.83*	37.50 ± 26.34*	43.49 ± 28.97*	− 6.63 (− 17.49 to 4.24)	− 3.93 (− 14.94 to 7.10)	− 2.70 (− 13.15 to 7.74)	0.34	− 0.19	− 0.009
Insomnia	37.06 ± 30.00	31.85 ± 25.58	33.32 ± 31.15	31.34 ± 30.37	27.51 ± 28.92	39.37 ± 34.52	− 10.17 (− 21.63 to 1.29)	− 11.17 (− 22.75 to 0.41)	1.00 (− 10.05 to 12.05)	0.04	− 0.39	− 0.37
Appetite loss	19.77 ± 29.00	24.50 ± 26.70	13.64 ± 22.17	13.33 ± 23.62*	19.53 ± 24.97	18.83 ± 26.66	− 7.08 (− 16.88 to 2.72)	− 3.45 (− 13.46 to 6.57)	− 3.64 (− 13.07 to 5.80)	0.22	− 0.44	− 0.41
Constipation	21.63 ± 27.96	21.45 ± 27.09	18.70 ± 26.87	10.14 ± 21.79*	12.39 ± 19.93*	14.47 ± 23.76	− 4.98 (− 13.23 to 3.29)	− 3.15 (− 11.51 to 5.20)	− 1.82 (− 9.77 to 6.12)	0.35	− 0.26	− 0.18
Diarrhea	14.52 ± 22.58	13.15 ± 22.87	15.78 ± 24.05	8.90 ± 18.88	17.74 ± 26.42	8.47 ± 16.55*	0.99 (− 7.58 to 9.55)	9.77 (1.09 to 18.44)*	− 8.78 (− 17.03 to − 0.53)*	0.01	0.07	0.51
Financial difficulties	21.73 ± 31.75	16.54 ± 26.85	20.11 ± 31.95	25.45 ± 34.71	23.19 ± 33.54*	18.67 ± 32.53	5.98 (− 4.96 to 16.92)	7.01 (− 4.07 to 18.08)	− 1.02 (− 11.58 to 9.53)	0.26	0.16	0.28

*RT–HIIT* resistance and high-intensity interval training, *AT–HIIT* moderate-intensity aerobic and high-intensity interval training, *UC* usual care, *EORTC-QLQ-C30* The European Organization for Research and Treatment of Cancer quality of life questionnaire

^†^ *p* values are for the interaction (ANCOVA adjusted for baseline values, chemotherapy, and menopausal status), * *p* < 0.05 pre- to post-intervention

### Changes in HRQoL

HRQoL measured by the EORTC-QLQ-C30 instrument showed a significant difference in role functioning in favor of RT–HIIT (ES = 0.81) and AT–HIIT (ES = 0.64) compared to declines for UC. Cognitive functioning was superior for RT–HIIT compared to declines reported by UC (ES = 0.35), while AT–HIIT significantly improved in emotional functioning compared to UC (ES = 0.40). The magnitude of negative change in physical functioning for RT–HIIT (ES = 0.49) and AT–HIIT (ES = 0.48) were significantly lower compared to UC. UC reported significantly higher pain scores compared to unchanged pain scores reported by AT–HIIT (ES = − 0.36) (Table 3).

### Changes in symptoms and symptom burden

A significant difference was found for symptom burden as measured by the MSAS, with RT–HIIT (ES = − 0.43) reporting a reduced symptom burden score and AT–HIIT (ES = − 0.42) was unchanged compared to an increased score for UC. The RT–HIIT group reported unchanged levels of total symptoms being significantly different compared to the increase in symptoms experienced by UC (ES = − 0.52) (Table 4).

**Table 4:** Symptoms (MSAS) outcome variables and change over 16 weeks

	Baseline Mean ± SD			16 weeks Mean ± SD			Adjusted mean change Mean (95% CI)			*p* value^†^	Effect size	
	RT–HIIT *n* = 74	AT–HIIT *n* = 70	UC *n* = 60	RT–HIIT *n* = 74	AT–HIIT *n* = 70	UC *n* = 60	RT–HIIT vs UC	AT–HIIT vs UC	RT–HIIT vs AT–HIIT		RT–HIIT vs UC	AT–HIIT vs UC
Symptom burden	0.91 ± 0.72	0.75 ± 0.58	0.70 ± 0.73	0.77 ± 0.60*	0.66 ± 0.55	0.87 ± 0.65*	− 0.22 (− 0.41 to − 0.02)*	− 0.24 (− 0.43 to − 0.04)*	0.02 (− 0.17 to 0.21)	< 0.01	− 0.43	− 0.42
Physical symptoms	0.74 ± 0.60^ϕ^	0.67 ± 0.49	0.51 ± 0.56	0.68 ± 0.55	0.75 ± 0.54	0.76 ± 0.59	− 0.18 (− 0.39 to − 0.02)	− 0.10 (− 0.31 to 0.11)	− 0.08 (− 0.28 to 0.11	0.10	− 0.56	− 0.39
Psychological symptoms	0.97 ± 0.76	0.81 ± 0.64	0.78 ± 0.77	1.02 ± 0.74	0.88 ± 0.69	1.08 ± 0.81	− 0.19 (− 0.43 to 0.05)	− 0.22 (− 0.46 to 0.03)	0.03 (− 0.21 to 0.26)	0.07	− 0.33	− 0.20
Total symptoms	0.74 ± 0.53	0.65 ± 0.41	0.58 ± 0.50	0.74 ± 0.49	0.76 ± 0.48*	0.84 ± 0.58*	− 0.20 (− 0.37 to − 0.04)*	− 0.13 (− 0.30 to 0.03)	− 0.07 (− 0.23 to 0.09)	0.01	− 0.52	− 0.36

*RT–HIIT* resistance and high-intensity interval training, *AT–HIIT* moderate-intensity aerobic and high-intensity interval training, *UC* usual care, *MSAS* Memorial Symptom Assessment Scale

^†^ *p* values are for the interaction (ANCOVA adjusted for baseline values, chemotherapy, and menopausal status), * *p* < 0.05 pre- to post-intervention, ^ϕ^ significant difference at baseline between RT–HIIT and UC

### Baseline status and changes in CRF, HRQoL, and symptom burden

**Fig. 2:**
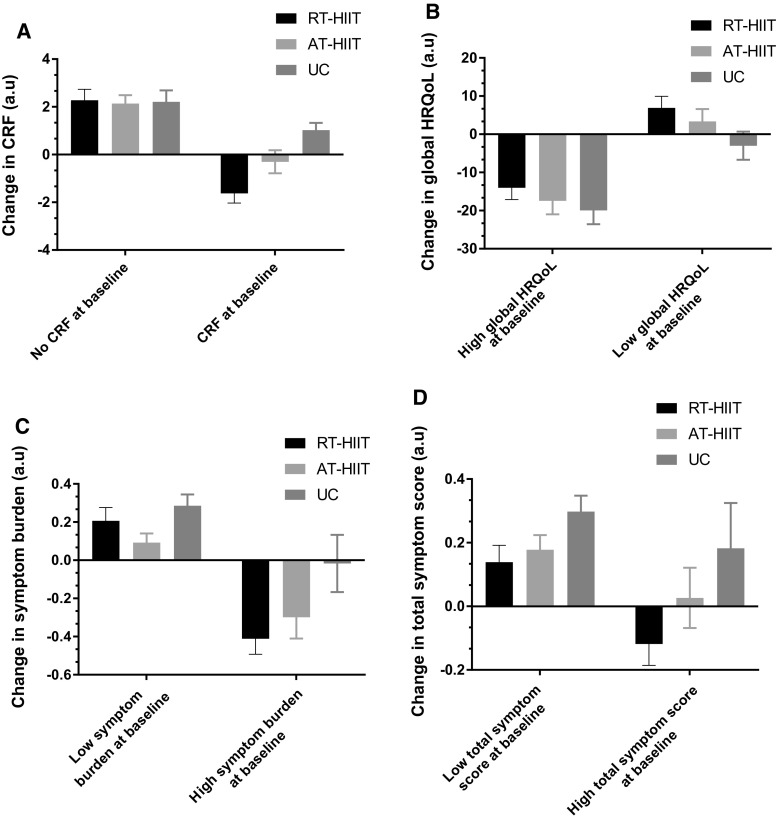
Baseline status and change after 16 weeks for the outcomes. **a** CRF (assessed by the Piper fatigue scale), **b** global HRQoL (assessed by the European Organization for Research and Treatment of cancer quality of life questionnaire), **c** symptom burden (assessed by the Memorial symptom assessment scale), and **d** total symptom score (assessed by the Memorial symptom assessment scale). *CRF* cancer-related fatigue, *HRQoL* health-related quality of life, *CRF* cancer-related fatigue, *HRQoL* health-related quality of life, *RT–HIIT* resistance and high-intensity interval training, *AT–HIIT* moderate-intensity aerobic and high-intensity interval training, *UC* usual care

Changes at 16 weeks for the outcomes CRF, global HRQoL, symptom burden, and total symptoms based on baseline status are shown in Fig. 2. In the sub-analysis, we show that, for the participants experiencing CRF at baseline, those in the UC group reported a further increase in CRF at the follow-up, while those in the exercise groups reported decreased levels of CRF. Similar results were shown for global HRQoL, symptom burden, and total symptom score where the participants exhibited beneficial effects from exercise training if they had reported a low global HRQoL or displayed a high symptom burden at baseline.

**Fig. 3:**
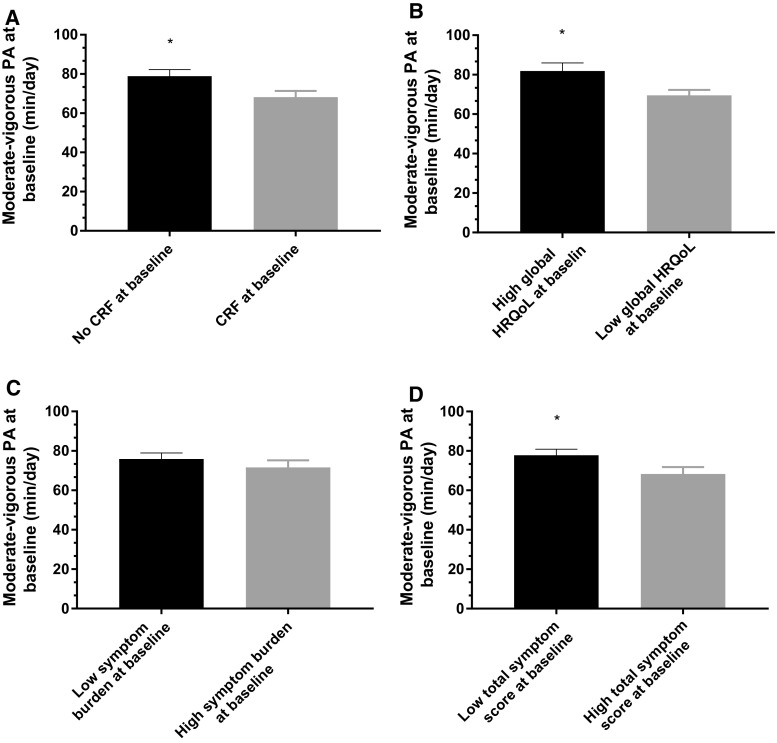
Activity levels at baseline based on the subgroups. **a** CRF and no CRF at baseline (assessed by the Piper fatigue scale), **b** low global HRQoL and high global HRQoL at baseline (assessed by the European Organization for Research and Treatment of cancer quality of life questionnaire), **c** high symptom burden and low symptom burden at baseline (assessed by the Memorial symptom assessment scale), and **d** high total symptom score and low total symptom score at baseline (assessed by the Memorial symptom assessment scale). *CRF* cancer-related fatigue, *HRQoL* health-related quality of life. *p < 0.05 between subgroups

At baseline, objectively assessed physical activity was significantly higher in groups reporting no CRF compared to those experiencing CRF (*p* = 0.02), with high global HRQoL levels compared to low global HRQoL at baseline (*p* = 0.01) and with a low total symptom score compared to high total symptom score at baseline (*p* = 0.04) (Fig. 3).

No adverse events were reported as a result of the testing or the exercise sessions. Of particular clinical importance, no adverse events were reported in relation to peripherally inserted central catheters (inserted in the majority of the participants’ arms) during the study period.

## Discussion

In the current trial, the effects of RT–HIIT, and aerobic and high-intensity interval training (AT–HIIT) compared to UC were assessed in women with breast cancer undergoing chemotherapy. We show that 16 weeks of RT–HIIT counteracts several dimensions of CRF and reduces symptom burden. Both RT–HIIT and AT–HIIT groups showed beneficial effects for vital aspects of HRQoL.

RT–HIIT counteracted increases in total CRF (ES = − 0.51), including physical (ES = − 0.48) and behavior/daily life aspects (ES = − 0.62) of CRF. However, no effects for cognitive or emotional CRF were found. This is in line with a recent meta-analysis [5] showing that not all dimensions of fatigue are affected by exercise training. Similar to our findings, a recent trial [39], including moderate- to high-intensity aerobic exercise and progressive resistance exercise, demonstrated that concurrent resistance and aerobic exercise training was effective in offsetting CRF, with an effect size of − 0.63 for physical CRF and − 0.29 for general CRF. Superior effects of moderate/high intensity was also shown compared to lower intensity, home-based exercise for physical CRF (ES = 0.42). In contrast, other trials [10, 40, 41] showed no difference in CRF between participants performing moderate-intensity combined aerobic and resistance training versus a control group, suggesting that higher intensity/load combined exercise training is required to counteract increases in CRF. Noteworthy, in the present study, the AT–HIIT group was not able to counteract an increase in multi-dimensional measures of CRF following the intervention period. However, results from the unidimensional EORTC-QLQ-C30 scale showed that participants in the AT–HIIT group were able to counteract CRF, similar to effects displayed by the RT–HIIT group, although the effect size for RT–HIIT was higher (− 0.61) compared to AT–HIIT (− 0.47). Only one previous trial [42] incorporating multi-modal training, which included high-intensity exercise training, has shown positive effects on CRF from the unidimensional EORTC-QLQ-C30 scale, leading us to, again, speculate that the beneficial and clinically relevant effects of exercise on CRF are intensity-dependent. The discrepant results for AT–HIIT when assessing multi-dimensional versus unidimensional fatigue are unclear. It may be that the PFS is not sensitive enough to detect clinically meaningful changes over time [27].

This is the first trial to demonstrate significant beneficial effects of exercise training on role and emotional functioning in patients with breast cancer during chemotherapy. The large effect size for the RT–HIIT group compared to UC for role functioning (ES = 0.81) suggests an important role for the resistance training component in particular. It has been shown that being involved in a supervised exercise program can improve both role and emotional functioning [43], likely through interactions with supervising personnel and peers in the exercise setting. Notably, only AT–HIIT improved in emotional functioning. Aerobic training has been proven superior to resistance training alone in improving emotional functioning in elderly [44]. Moreover, aerobic exercise has been shown to decrease depressive and anxiety symptoms in breast cancer survivors [45]. An increased level of self-efficacy has been suggested as a possible mechanism for improved emotional functioning in elderly engaged in exercise [43], which in this case may be linked to feelings of being able to carry out the exercises and feeling more revitalized after the exercise sessions.

Results from the current trial demonstrated that RT–HIIT counteracted increases in self-reported cognitive problems. The effect size of 0.35 is comparable to one previous trial that included high-intensity exercise, with a similar effect size (0.32) [39], while other trials [42, 46, 47] have failed to show beneficial effects of exercise on cognitive functioning. Once more, we speculate that intensity may play a crucial role in offsetting a lowered HRQoL. Cognitive dysfunction experienced during chemotherapy is associated with psychological distress and persists for several years post chemotherapy [48]. Pain is another debilitating symptom caused by chemotherapy. In the current trial, the AT–HIIT group experienced significantly less pain compared to the UC group. A substantial number of patients receiving anthracycline/taxane-based chemotherapy experience pain, particularly during the taxane component [49] that is not relieved by analgesics [50]. Therefore, it is of great importance to implement successful interventions, such as high-intensity training, at an early stage in the survivorship continuum to aid in symptom relief.

Our results demonstrate that exercise training during chemotherapy is effective to minimize the burden of symptoms related to chemotherapy. Few exercise trials have reported on symptom burden among women with breast cancer [45]. One consequence of a high degree of symptom burden may be chemotherapy dose reductions or even discontinued treatment, which in turn may influence prognosis [51]. In fact, two previous trials [10, 39] showed the potential of exercise to improve chemotherapy completion rates, and a trial by Courneya et al. [52] demonstrated that adding exercise to standard chemotherapy may improve breast cancer outcomes. With an increasing breast cancer survival rate, a larger number of women will experience different long-term side effects [2], including symptoms related to chemotherapy, which not only affects individuals’ HRQoL negatively, but also aspects of daily life involving family and work performance [53]. Given the findings from this trial showing the effectiveness of exercise for managing symptom burden, it is of great importance to support patients to be physically active during treatment in order to prevent a progressive downward spiral in declining health for these patients.

In concordance with a recent trial [54], in a sub-analysis, we found that the participants with the highest levels of fatigue and symptoms and the lowest HRQoL were those who gained the greatest benefits from the exercise training. Importantly, participants with higher levels of CRF, symptoms, and lower HRQoL were also significantly less active than those with lower levels of CRF, symptoms, and a better HRQoL at baseline. It is therefore likely that beneficial effects from any exercise training regimens are dependent on fitness status at baseline.

Strengths of the OptiTrain trial are the RCT design, that two types of supervised exercise regimens were trialed during chemotherapy, the use of validated instruments, and a large sample size. Limitations of the study include the high dropout rate by the UC patients directly following randomization that may have led to a selection bias. This aspect should generally be considered when drawing conclusions from exercise intervention studies. While our recruitment and attendance rates are within the range commonly reported in exercise trials [55], supporting participants to complete exercise programs as prescribed remains a challenge for the field of exercise oncology.

## Conclusion

In conclusion, results from the OptiTrain study show that a training program including RT–HIIT is effective to counteract both multi- and unidimensional aspects of CRF and to reduce symptom burden, in women with breast cancer during chemotherapy. Both RT–HIIT and AT–HIIT were effective to improve or maintain vital aspects of HRQoL. Findings from the OptiTrain trial add important evidence on benefits of high-intensity interval training for health outcomes for patients during chemotherapy, allowing health professionals in the oncology setting to recommend adding high-intensity interval exercise to specific and targeted programs for patients with breast cancer.

## References

[1] Peto R, Davies C, Godwin J, Gray R, Pan HC, Clarke M, Cutter D, Darby S, McGale P, Taylor C, Wang YC, Bergh J, Di Leo A, Albain K, Swain S, Piccart M, Pritchard K (2012). Comparisons between different polychemotherapy regimens for early breast cancer: meta-analyses of long-term outcome among 100,000 women in 123 randomised trials. Lancet (Lond, Engl).

[2] Barbaric M, Brooks E, Moore L, Cheifetz O (2010). Effects of physical activity on cancer survival: a systematic review. Physiother Can.

[3] Davies NJ, Batehup L, Thomas R (2011). The role of diet and physical activity in breast, colorectal, and prostate cancer survivorship: a review of the literature. Br J Cancer.

[4] Bower JE (2014). Cancer-related fatigue-mechanisms, risk factors, and treatments. Nat Rev Clin Oncol.

[5] van Vulpen JK, Peeters PH, Velthuis MJ, van der Wall E, May AM (2016). Effects of physical exercise during adjuvant breast cancer treatment on physical and psychosocial dimensions of cancer-related fatigue: a meta-analysis. Maturitas.

[6] Medysky ME, Temesi J, Culos-Reed SN, Millet GY (2017). Exercise, sleep and cancer-related fatigue: are they related?. Clin Neurophysiol.

[7] Casla S, Lopez-Tarruella S, Jerez Y, Marquez-Rodas I, Galvao DA, Newton RU, Cubedo R, Calvo I, Sampedro J, Barakat R, Martin M (2015). Supervised physical exercise improves VO_2max_, quality of life, and health in early stage breast cancer patients: a randomized controlled trial. Breast Cancer Res Treat.

[8] Meneses-Echavez JF, Gonzalez-Jimenez E, Ramirez-Velez R (2015). Supervised exercise reduces cancer-related fatigue: a systematic review. J Physiother.

[9] Mustian KM, Alfano CM, Heckler C (2017). Comparison of pharmaceutical, psychological, and exercise treatments for cancer-related fatigue: a meta-analysis. JAMA Oncol.

[10] Courneya KS, Segal RJ, Mackey JR, Gelmon K, Reid RD, Friedenreich CM, Ladha AB, Proulx C, Vallance JK, Lane K, Yasui Y, McKenzie DC (2007). Effects of aerobic and resistance exercise in breast cancer patients receiving adjuvant chemotherapy: a multicenter randomized controlled trial. J Clin Oncol.

[11] Schmitz KH, Courneya KS, Matthews C, Demark-Wahnefried W, Galvão DA, Pinto BM, Irwin ML, Wolin KY, Segal RJ, Lucia A, Schneider CM, von Gruenigen VE, Schwartz AL, Medicine A (2010). American College of Sports Medicine roundtable on exercise guidelines for cancer survivors. Med Sci Sports Exerc.

[12] Tjonna AE, Lee SJ, Rognmo O, Stolen TO, Bye A, Haram PM, Loennechen JP, Al-Share QY, Skogvoll E, Slordahl SA, Kemi OJ, Najjar SM, Wisloff U (2008). Aerobic interval training versus continuous moderate exercise as a treatment for the metabolic syndrome: a pilot study. Circulation.

[13] Wisloff U, Stoylen A, Loennechen JP, Bruvold M, Rognmo O, Haram PM, Tjonna AE, Helgerud J, Slordahl SA, Lee SJ, Videm V, Bye A, Smith GL, Najjar SM, Ellingsen O, Skjaerpe T (2007). Superior cardiovascular effect of aerobic interval training versus moderate continuous training in heart failure patients: a randomized study. Circulation.

[14] Jaureguizar KV, Vicente-Campos D, Bautista LR, de la Pena CH, Gomez MJ, Rueda MJ, Fernandez Mahillo I (2016). Effect of high-intensity interval versus continuous exercise training on functional capacity and quality of life in patients with coronary artery disease: a randomized clinical trial. J Cardiopulm Rehabil Prev.

[15] Thum JS, Parsons G, Whittle T, Astorino TA (2017). High-intensity interval training elicits higher enjoyment than moderate intensity continuous exercise. PLoS ONE.

[16] Ouerghi N, Selmi O, Ben Khalifa W, Ben Fradj MK, Feki M, Kaabachi N, Bouassida A (2016). Effect of high-intensity intermittent training program on mood state in overweight/obese young men. Iran J Public Health.

[17] Drigny J, Gremeaux V, Dupuy O, Gayda M, Bherer L, Juneau M, Nigam A (2014). Effect of interval training on cognitive functioning and cerebral oxygenation in obese patients: a pilot study. J Rehabil Med.

[18] Saanijoki T, Tuominen L, Tuulari JJ, Nummenmaa L, Arponen E, Kalliokoski K, Hirvonen J (2017). Opioid release after high-intensity interval training in healthy human subjects. Neuropsychopharmacology.

[19] Schmitt J, Lindner N, Reuss-Borst M, Holmberg HC, Sperlich B (2016). A 3-week multimodal intervention involving high-intensity interval training in female cancer survivors: a randomized controlled trial. Physiol Rep.

[20] Dolan LB, Campbell K, Gelmon K, Neil-Sztramko S, Holmes D, McKenzie DC (2016). Interval versus continuous aerobic exercise training in breast cancer survivors: a pilot RCT. Support Care Cancer.

[21] Schulz SVW, Laszlo R, Otto S, Prokopchuk D, Schumann U, Ebner F, Huober J, Steinacker JM (2017). Feasibility and effects of a combined adjuvant high-intensity interval/strength training in breast cancer patients: a single-center pilot study. Disabil Rehabil.

[22] Green N, Wertz T, LaPorta Z, Mora A, Serbas J, Astorino TA (2017). Comparison of acute physiological and psychological responses between moderate intensity continuous exercise and three regimes of high intensity training. J Strength Cond Res.

[23] Wengström Y, Bolam KA, Mijwel S, Sundberg CJ, Backman M, Browall M, Norrbom J, Rundqvist H (2017). Optitrain: a randomised controlled exercise trial for women with breast cancer undergoing chemotherapy. BMC Cancer.

[24] Börjesson M, Urhausen A, Kouidi E, Dugmore D, Sharma S, Halle M, Heidbuchel H, Björnstad HH, Gielen S, Mezzani A, Corrado D, Pelliccia A, Vanhees L (2011). Cardiovascular evaluation of middle-aged/senior individuals engaged in leisure-time sport activities: position stand from the sections of exercise physiology and sports cardiology of the European Association of Cardiovascular Prevention and Rehabilitation. Eur J Cardiovasc Prev Rehabil.

[25] Borg GA (1982). Psychophysical bases of perceived exertion. Med Sci Sports Exerc.

[26] Mayhew JL, Prinster JL, Ware JS, Zimmer DL, Arabas JR, Bemben MG (1995). Muscular endurance repetitions to predict bench press strength in men of different training levels. J Sports Med Phys Fitness.

[27] Jakobsson S, Taft C, Östlund U, Ahlberg K (2013). Performance of the Swedish version of the Revised Piper Fatigue Scale. Eur J Oncol Nurs.

[28] Piper BF, Dibble SL, Dodd MJ, Weiss MC, Slaughter RE, Paul SM (1998). The revised Piper Fatigue Scale: psychometric evaluation in women with breast cancer. Oncol Nurs Forum.

[29] Aaronson NK, Ahmedzai S, Bergman B, Bullinger M, Cull A, Duez NJ, Filiberti A, Flechtner H, Fleishman SB, de Haes JC (1993). The European Organization for Research and Treatment of Cancer QLQ-C30: a quality-of-life instrument for use in international clinical trials in oncology. J Natl Cancer Inst.

[30] Minton O, Stone P (2009). A systematic review of the scales used for the measurement of cancer-related fatigue (CRF). Ann Oncol.

[31] Uwer L, Rotonda C, Guillemin F, Miny J, Kaminsky MC, Mercier M, Tournier-Rangeard L, Leonard I, Montcuquet P, Rauch P, Conroy T (2011). Responsiveness of EORTC QLQ-C30, QLQ-CR38 and FACT-C quality of life questionnaires in patients with colorectal cancer. Health Qual Life Outcomes.

[32] Portenoy RK, Thaler HT, Kornblith AB, Lepore JM, Friedlander-Klar H, Kiyasu E, Sobel K, Coyle N, Kemeny N, Norton L (1994). The Memorial Symptom Assessment Scale: an instrument for the evaluation of symptom prevalence, characteristics and distress. Eur J Cancer (Oxford, England: 1990).

[33] Browall M, Sarenmalm EK, Nasic S, Wengstrom Y, Gaston-Johansson F (2012). Validity and reliability of the Swedish Version of the Memorial Symptom Assessment Scale (MSAS): an instrument for the evaluation of symptom prevalence, characteristics, and distress. J Pain Symptom Manage.

[34] Snyder CF, Blackford AL, Brahmer JR, Carducci MA, Pili R, Stearns V, Wolff AC, Dy SM, Wu AW (2010). Needs assessments can identify scores on HRQOL questionnaires that represent problems for patients: an illustration with the Supportive Care Needs Survey and the QLQ-C30. Qual Life Res.

[35] Aguilar-Farias N, Brown WJ, Peeters GM (2014). ActiGraph GT3X + cut-points for identifying sedentary behaviour in older adults in free-living environments. J Sci Med Sport.

[36] Morris SB (2007). Estimating effect sizes from pretest-posttest-control group designs. Organ Res Methods.

[37] Cohen J (1988). Statistical power analysis for the behavioral sciences.

[38] Rubin LH, Witkiewitz K, Andre JS, Reilly S (2007). Methods for handling missing data in the behavioral neurosciences: don’t throw the baby rat out with the bath water. JUNE.

[39] van Waart H, Stuiver MM, van Harten WH, Geleijn E, Kieffer JM, Buffart LM, de Maaker-Berkhof M, Boven E, Schrama J, Geenen MM, Meerum Terwogt JM, van Bochove A, Lustig V, van den Heiligenberg SM, Smorenburg CH, Hellendoorn-van Vreeswijk JA, Sonke GS, Aaronson NK (2015). Effect of low-intensity physical activity and moderate- to high-intensity physical exercise during adjuvant chemotherapy on physical fitness, fatigue, and chemotherapy completion rates: results of the PACES Randomized Clinical Trial. J Clin Oncol.

[40] Mutrie N, Campbell AM, Whyte F, McConnachie A, Emslie C, Lee L, Kearney N, Walker A, Ritchie D (2007). Benefits of supervised group exercise programme for women being treated for early stage breast cancer: pragmatic randomised controlled trial. BMJ.

[41] Campbell A, Mutrie N, White F, McGuire F, Kearney N (2005). A pilot study of a supervised group exercise programme as a rehabilitation treatment for women with breast cancer receiving adjuvant treatment. Eur J Oncol Nurs.

[42] Adamsen L, Quist M, Andersen C, Moller T, Herrstedt J, Kronborg D, Baadsgaard MT, Vistisen K, Midtgaard J, Christiansen B, Stage M, Kronborg MT, Rorth M (2009). Effect of a multimodal high intensity exercise intervention in cancer patients undergoing chemotherapy: randomised controlled trial. BMJ.

[43] McAuley E, Blissmer B, Katula J, Duncan TE, Mihalko SL (2000). Physical activity, self-esteem, and self-efficacy relationships in older adults: a randomized controlled trial. Ann Behav Med.

[44] Penninx BW, Rejeski WJ, Pandya J, Miller ME, Di Bari M, Applegate WB, Pahor M (2002). Exercise and depressive symptoms: a comparison of aerobic and resistance exercise effects on emotional and physical function in older persons with high and low depressive symptomatology. J Gerontol Ser B.

[45] Furmaniak AC, Menig M, Markes MH (2016). Exercise for women receiving adjuvant therapy for breast cancer. Cochrane Database Syst Rev.

[46] Schmidt T, Weisser B, Durkop J, Jonat W, Van Mackelenbergh M, Rocken C, Mundhenke C (2015). Comparing endurance and resistance training with standard care during chemotherapy for patients with primary breast cancer. Anticancer Res.

[47] Travier N, Velthuis MJ, Steins Bisschop CN, van den Buijs B, Monninkhof EM, Backx F, Los M, Erdkamp F, Bloemendal HJ, Rodenhuis C, de Roos MA, Verhaar M, ten Bokkel Huinink D, van der Wall E, Peeters PH, May AM (2015). Effects of an 18-week exercise programme started early during breast cancer treatment: a randomised controlled trial. BMC Med.

[48] Lyon DE, Cohen R, Chen H, Kelly DL, Starkweather A, Ahn HC, Jackson-Cook CK (2016). The relationship of cognitive performance to concurrent symptoms, cancer- and cancer-treatment-related variables in women with early-stage breast cancer: a 2-year longitudinal study. J Cancer Res Clin Oncol.

[49] Saibil S, Fitzgerald B, Freedman OC, Amir E, Napolskikh J, Salvo N, Dranitsaris G, Clemons M (2010). Incidence of taxane-induced pain and distress in patients receiving chemotherapy for early-stage breast cancer: a retrospective, outcomes-based survey. Curr Oncol.

[50] van Helmond N, Steegers MA, Filippini-de Moor GP, Vissers KC, Wilder-Smith OH (2016). Hyperalgesia and persistent pain after breast cancer surgery: a prospective randomized controlled trial with perioperative COX-2 inhibition. PLoS ONE.

[51] Stasi R, Abriani L, Beccaglia P, Terzoli E, Amadori S (2003). Cancer-related fatigue: evolving concepts in evaluation and treatment. Cancer.

[52] Courneya KS, Segal RJ, McKenzie DC, Dong H, Gelmon K, Friedenreich CM, Yasui Y, Reid RD, Crawford JJ, Mackey JR (2014). Effects of exercise during adjuvant chemotherapy on breast cancer outcomes. Med Sci Sports Exerc.

[53] Wu HS, Harden JK (2015). Symptom burden and quality of life in survivorship: a review of the literature. Cancer Nurs.

[54] Taaffe DR, Newton RU, Spry N, Joseph D, Chambers SK, Gardiner RA, Wall BA, Cormie P, Bolam KA, Galvao DA (2017). Effects of different exercise modalities on fatigue in prostate cancer patients undergoing androgen deprivation therapy: a year-long randomised controlled trial. Eur Urol.

[55] Carayol M, Delpierre C, Bernard P, Ninot G (2015). Population-, intervention- and methodology-related characteristics of clinical trials impact exercise efficacy during adjuvant therapy for breast cancer: a meta-regression analysis. Psycho-oncology.

